# Anxiety and depression symptoms and migraine: a symptom-based approach research

**DOI:** 10.1186/s10194-017-0742-1

**Published:** 2017-03-21

**Authors:** Mario Fernando Prieto Peres, Juliane P. P. Mercante, Patricia R. Tobo, Helder Kamei, Marcelo Eduardo Bigal

**Affiliations:** 1 Albert Einstein Hospital, R Joaquim Eugenio de Lima, 881 cj 709, 01403-001 São Paulo, SP Brazil; 2 Natura, São Paulo, Brazil; 3 Teva Pharmaceuticals, North Wales, PA USA; 4 Institute of Psychiatry, University of São Paulo, São Paulo, Brazil

**Keywords:** Migraine, Anxiety, Depression, Comorbidity

## Abstract

**Background:**

Anxiety and mood disorders have been shown to be the most relevant psychiatric comorbidities associated with migraine, influencing its clinical course, treatment response, and clinical outcomes. Limited information is available on how specific anxiety and depression symptoms are related to migraine. Symptoms-based approach, a current trend in mental health research, may improve our understanding in migraine comorbidity. The purpose of this study was to analyze how anxiety and depression aspects are related to migraine through a symptom-based approach.

**Methods:**

We studied 782 patients from the general population who completed a self-administered questionnaire assessing demographics, headache features, anxiety and depression symptoms. A binary logistic regression analyses were conducted to test the association between all four ratings in GAD-7 (anxiety) and PHQ-9 (depression) scales subitems as covariates, and migraine vs no headache as the outcome.

**Results:**

The leading Odd Ratios (OR) observed in individuals with migraine relative to those without migraine were anxiety related, “Not being able to stop or control worrying” on a daily basis [OR (CI 95%)] 49.2 (13.6–178.2), “trouble relaxing” 25.7 (7.1–92.6), “Feeling nervous, anxious or on edge” on a daily basis 25.4 (6.9–93.8), and “worrying too much about different things” 24.4 (7.7–77.6). Although the hallmark symptoms of depression are emotional (hopelessness and sadness), the highest scores found were physical: apetite, fatigue, and poor sleep. Irritability had a significant increase in migraine risk [OR 3.8 (1.9–7.8) if experienced some days, 7.5 (2.7–20.7) more than half the days, and 22.0 (5.7–84.9) when experienced nearly every day].

**Conclusions:**

Anxiety was more robustly associated with increase in migraine risk than depression. Lack of ability to properly control worrying and to relax are the most prominent issues in migraine psychiatric comorbidity. Physical symptoms in depression are more linked to migraine than emotional symptoms. A symptom-based approach helps clarifying migraine comorbidity and should be replicated in other studies.

## Background

Migraine is known to be a multifactorial disorder, with genetic, hormonal, environmental, dietary, sleep and psychological aspects playing a different role in each individual [1], it is considered a bio-behavioural disorder [2].

Anxiety and mood disorders have been shown to be the most relevant psychiatric comorbidities associated with migraine, influencing disease prevalence, prognosis, treatment and clinical outcomes [3]. Studies reported that mood and anxiety disorders are two to ten times more common in migraineurs than in the general population [4]. Furthermore, they are related, both at the clinical settings [5] and general population [6], to poorer quality of life [7] and increased suicide risk [8]. In addition, psychiatric comorbidities are risk factors for the progression from episodic to chronic migraine [9]. Patients with tension type headache and comorbid psychiatric disorders often exhibit affective temperament dysregulation and suicidal behaviors [10, 11].

Anxiety and mood disorders coexist in the general population at large and also in headache patients [5]. The hallmark of anxiety is excessive of worry, while in depression lack of energy, motivation and sadness prevail. Both disorders display significant physical symptoms. In anxiety, irritability, concentration problems, and agitation are common, whereas in depression, fatigue, concentration, sleep and appetite are included in the diagnostic definition [12]. Although anxiety and mood disorders are consolidated as the two main psychological issues related to migraine, limited information is available on what symptoms or aspects are more relevant. A more detailed study looking into these variables is necessary.

Our objective was to test the hypothesis that migraine might be not equally related to anxiety and mood symptoms, so we designed a symptom-based approach study, a current trend in mental health research, to answer this question.

## Methods

### Subjects

This was a population-based, cross-sectional study conducted from August to October 2014 in a representative sample of the Brazilian population and drawn from a panel maintained by Qualtrics. Participants were invited by e-mail to complete a self-administered, online survey, in return for incentives/cash honorarium (as established a priori within the panels’ agreements). As surveys were being completed, response patterns were monitored against established quotas and made decisions about sampling in order to meet them. Quotas have been set in order to limit the respondents according to social class distribution, age, gender, geographic location so the population surveyed could meet the same profile of the general adult population in Brazil, according to the 2010 census.

Security checks and quality verifications were used on all sources before respondents had begun the survey. Participants were included if they had online access to the Internet and were excluded if their email was no longer valid, if they did not answered or incomplete answered the questionnaire or if they did not accept the consent term. Quality checks questions and attention filters were added. Questions were divided into five blocks randomized so the impact of tiredness of respondents affected equally all questions. Force response validation was included in all questions. The length of interview was less than 30 min.

In order to evaluate how much the online population would differ from a general population, the same questionnaire was completed by a door-to-door population of 201 randomly assigned individuals in the city of São Paulo (Brazil). No differences between groups were found in education (*p* = 0.793), income (*p* = 0.078), BMI (*p* = 0.662) and gender (*p* = 0.313), but age in the online sample was younger (34.5 vs. 39.8, *p* < 0.001). All participants provided written consent. The protocol was approved by the Albert Einstein Hospital ethics committee.

### Instruments

Respondents were asked to complete a self-administered questionnaire assessing the following sociodemographics: gender, age, family income, self-reported weight and height, marital status and education. They were also asked about symptoms necessary to assign a migraine diagnoses as per the International Classification of Headache Disorders, second edition (ICHD-2) criteria [13].

Anxiety was assessed with the “General Anxiety Disorder-7” (GAD-7) scale; while depression was estimated with the “Patient Health Questionnaire-9” (PHQ-9) scale. Data on religiousness, optimism, life satisfaction, and happiness were analyzed elsewhere.

#### Anxiety

GAD-7 [14] is a questionnaire designed to assess anxiety symptoms, patients are invited to respond to 7 questions assessing a two-weeks period. Questions are : A) Feeling nervous, anxious or on edge; B) Not being able to stop or control worrying; worrying too much about different things; C) Trouble relaxing; D) Being so restless that is hard to sit still, E) becoming easily annoyed or irritable, F) Feeling afraid as if something awful might happen. Four alternatives are offered: 1. Not at all, 2. Several days, 3. More than half the days, and 4. Nearly every day. Scores can range from 0 to 21. The scale has been validated to portuguese [15].

#### Depression

The PHQ-9 is a 9-item scoring scale designed and validated for diagnosis and grading depression based on DSM-IV criteria, including the following aspects : (1) anhedonia; (2) depressed mood; (3) trouble sleeping; (4) feeling tired; (5) change in appetite; (6) guilt, self-blame, or worthlessness; (7) trouble concentrating; (8) feeling slowed down or restless; and (9) thoughts of being better off dead or hurting oneself [16]. Symptoms are rated using a 4-point scale (0 - never; 1 – several days; 2 - more than half the time; and 3 - nearly every day) regarding the past two weeks experienced. The overall scores ranged from 0 to 27. Validation to portugueses is available [17].

Participants were not tested for psychiatric conditions and data regarding psychoactive substances were not collected.

### Statistical analysis

Sample size calculation was done based on previous studies, we estimated a difference in 10% between migraine and controls in anxiety and depression symptoms, with a 5% significancy and power of 80%, a target of 209 patients were necessary.

Binary logistic regression was applied [18]. Covariates were items in GAD-7 and PHQ-9 scales, and outcome was migraine vs no headache in the past year, never reporting migraine in life. Control group had no symptoms of headache, and was free from any reported complicating medical or psychiatric conditions. Only the online population was used in the analysis.

Univariate binary logistic regression analyses were conducted to test the association between each covariate and the outcome [19]. All four ratings in GAD-7 and PHQ-9 subitems were analyzed as covariates. Sociodemographic variables were included and the results adjusted accordingly. The assumption of linearity in the logit scale (log-odds) between each quantitative covariate and the outcome in binary logistic regression analysis was assessed by examination of smoothed scatter plots. A multiple test correction with Bonferroni procedure was performed. The software R (R Foundation for Statistical Computing, Vienna, Austria) [20] was used for statistical analyses. A *p*-value of <0.05 was considered to be statistically significant, and all reported p-values were two-sided.

## Results

**Fig. 1:**
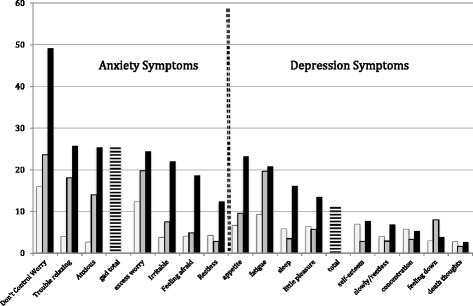
Odds ratios for migraine risk according to anxiety (*left*) and depression (*right*) symptoms

From 967 participants reached, 782 (80.8%) completed the questionnaire. The main reason for uncompleted responses was availability of less than 70% of valid or finished answers. Participants were women in 51.0%, mean age of 34.4years (SD: 11.3), married (51.5%), with university level education – completed or in course (50.6%) and with an income of more than US$400.00 (65.8%). 213 patients fit diagnostic criteria for migraine. Table 1 describes migraine characteristics. Tables 2 and 3 shows odds ratios and confidence intervals for GAD-7 and PHQ-9 subitems as covariates in univariate logistic regression 1 is the compared aspect wich is none in the aspect, 2 is some, 3 is moderate, 4 is severe. Binary outcomes migraine versus no pain controls. Figure 1 shows GAD-7 and PHQ-9 subitens ORs with ratings 4, 3, 2 versus 1 (none).

**Table 1:** Migraine patients demographics and clinical characteristics

	Migraine
Demographics	N = 213
Age, Mean (SD), y	34.2 (6.3)
Gender, women, %	73%
Race (%)	
White	61
Black	6
Asian	2
Pardo (Mixed)	31
Education Level (%)	
Primary	8
Secondary	61
University	31
BMI, mean (SD)	26.1 (5.4)
Marital Status	
Single	25
Separated/Divorced	8
Married	67
Headache characteristics	
Frequency, Mean (SD)	6.5 (3.7)
Frequency > = 15 days/month (%)	18
History, Mean (SD), years	21.1 (8.2)
Intensity, Mean (SD), 0-10 scale	3.9 (2.2)
Midas Grade (%)	
I	28
II	15
III	25
IV	32

**Table 2:** Univariate logistic regression for depression (PHQ-9) aspects as covariates and migraine vs no headache as binary outcome

Covariate	Score Level 1 = reference	N	Odds ratio (95% CI)	*P*-value
Depression				
Little interest or pleasure in doing things	1	61	-	-
	2	115	6.42 (3.12–13.20)	**<.001**
	3	23	5.74 (2.03–16.21)	**<.001**
	4	14	13.54 (3.29–55.79)	**<.001**
Feeling down, depressed, or hopeless	1	75	-	**-**
	2	97	3.03 (1.61–5.69)	**<.001**
	3	24	8.07 (2.69–24.21)	**<.001**
	4	17	3.90 (1.29–11.78)	**0.016**
Trouble falling or staying asleep, or sleeping too much	1	72	-	**-**
	2	88	5.95 (2.96–11.95)	**<.001**
	3	23	3.53 (1.32–9.43)	**0.012**
	4	30	16.17 (5.37–48.76)	**<.001**
Feeling tired or having little energy	1	53	-	**-**
	2	99	9.30 (3.82–22.64)	**<.001**
	3	36	19.71 (6.59–58.99)	**<.001**
	4	25	20.81 (6.18–70.09)	**<.001**
Poor appetite or overeating	1	68	-	**-**
	2	83	6.74 (3.19–14.26)	**<.001**
	3	36	9.61 (3.79–24.41)	**<.001**
	4	26	23.26 (6.83–79.17)	**<.001**
Feeling bad about yourself	1	95	-	**-**
	2	71	7.05 (3.52–14.09)	**<.001**
	3	26	2.79 (1.15–6.79)	**0.023**
	4	21	7.66 (2.56–22.93)	**<.001**
Trouble concentrating	1	92	-	**-**
	2	74	5.77 (2.94–11.30)	**<.001**
	3	27	3.32 (1.37–8.07)	**0.008**
	4	20	5.33 (1.86–15.31)	**0.002**
Moving or speaking slowly Or restless	1	114	-	**-**
	2	57	4.03 (2.04–7.99)	**<.001**
	3	27	2.91 (1.22–6.95)	**0.016**
	4	15	6.86 (1.83–25.70)	**0.004**
Death thoughts	1	153	-	**-**
	2	31	2.82 (1.22–6.53)	**0.015**
	3	19	1.59 (0.61–4.17)	0.347
	4	10	2.69 (0.67–10.81)	0.162

**Table 3:** Univariate logistic regression for anxiety (GAD-7) aspects as covariates and migraine vs no headache as binary outcome

Covariate	Score level	N	Odds ratio (95% CI)	*P*-value
Anxiety				
Feeling nervous, anxious or on edge	1	36	-	-
	2	114	2.73 (1.15–6.52)	0.023
	3	30	14.00 (4.26–46.05)	<.001
	4	33	25.37 (6.86–93.83)	<.001
Not being able to stop or control worrying	1	71	-	-
	2	85	16.03 (6.76–38.02)	<.001
	3	28	23.62 (7.65–73.00)	<.001
	4	29	49.22 (13.60–178.18)	<.001
Worrying too much about different things	1	59	-	-
	2	91	12.45 (5.08–30.51)	<.001
	3	33	19.81 (6.60–59.50)	<.001
	4	30	24.41 (7.68–77.62)	<.001
Trouble relaxing	1	46	-	-
	2	111	4.04 (1.78–9.15)	<.001
	3	27	18.09 (5.37–60.90)	<.001
	4	29	25.69 (7.13–92.64)	<.001
Being so restless that is hard to sit still				
	1	98	-	-
	2	68	4.31 (2.23–8.35)	<.001
	3	26	2.81 (1.16–6.82)	0.022
	4	21	12.37 (3.39–45.06)	<.001
Becoming easily annoyed or irritable	1	58	-	-
	2	104	3.81 (1.87–7.79)	<.001
	3	27	7.46 (2.69–20.74)	<.001
	4	24	22.00 (5.70–84.95)	<.001
Feeling afraid as if something awful migth happen	1	99	-	-
	2	69	4.11 (2.14–7.90)	<.001
	3	26	4.94 (1.94–12.57)	<.001
	4	19	18.65 (4.06–85.72)	<.001

All anxiety and depression items were significantly related to migraine compared to non-headache controls (*p* < 0.05), with the exception of death thoughts at the two higher ratings.

When looking at anxiety and depression symptoms and their relation to migraine, odds ratio are much higher for anxiety symptoms than for depressive symptoms (Table 2). Anxiety items had increasingly higher odds, meanignn that the higher the score, the higher the odds for migraine. This was not seen for the majority of depression symptoms. The leading ORs observed were anxiety related, “Not being able to stop or control worrying” on a daily basis [OR (CI 95%)] 49.2 (13.6–178.2), “trouble relaxing” 25.7 (7.1–92.6), “Feeling nervous, anxious or on edge” on a daily basis 25.4 (6.8–93.8), and “worrying too much about different things” 24.4 (7.6–77.6).

Depressive symptoms scored the highest odds were physical symptoms, first “poor appetite or overeating” [OR (CI 95%)] 6.74 (3.2–14.3) for some days, 9.6 (3.8–24.4) when experienced more than half the days, and 23.3 (6.8–79.2) when experienced nearly every day. “Feeling tired or having little energy was 9.30 (3.8–22.6), 19.7 (6.6–59.0), and 20.8 (6.2–70.1); “trouble falling or staying asleep or sleeping too much” 5.9 (2.9–11.9), 3.5 (1.3–9.4), 16.2 (5.4–48.8).

## Discussion

The results presented in this paper provide information on the relation between anxiety, depression and migraine. It shows higher odds in anxiety than depression, and found some inner aspects of anxiety and depression more important than others. Previously published articles showed significant implication of depression and anxiety in migraine, but which aspects, domains or symptoms are more relevant have not been studied in great detail. A recent study focused on affective disorders symptoms in migraineurs [3] analysing patients from different Dutch databases, LUMINA (migraine) and NESDA (depression and anxiety), using the Mood and Anxiety Questionnaire (MASQ-30) to assess three dimensions —lack of positive affect (depression specific); negative affect (nonspecific); and somatic arousal (anxiety specific), the later being the most important.

Specific aspects or symptoms in depression and/or anxiety may be more relevant for migraineurs, being more sensitive than non-migraine headache sufferers, or non-headache pain, or non-pain individuals in developing a certain clinical psychopathological presentation. Our data supports the idea of anxiety being more implicated to migraine than depression.

When analyzing sub-items utilizing the first rating (not at all) versus the second possible rating or the first positive rating (some days) as covariables, (Tables 2 and 3), even mild symptomatology were significantly related to migraine with the exception of thoughts about dying.

One may consider not only a GAD DSM diagnosis, but a trait, or a minimal amount of worry, inability to control anxiety symptoms, feeling afraid, nervous, or anxious can play a critical role in migraine, by triggering an attack, making it lasting longer, affecting headache frequency and duration [21, 22] quality of life, health care expenditures [23] and chronification [9]. In a Brazilian population, we found anxiety subthreshold and full DSM diagnosis (GAD) predominantly affecting migraine risk, generalized anxiety disorder (GAD) with [OR (CI 95%)] 7.0 (4.2–11.7) for all primary headaches, 7.8 (4.3–14.1) for migraine, 12.8 (4.5–36.3) for chronic migraine, and 3.9 (1.3–11.9) for tension-type headache. Subthreshold anxiety showed significantly higher ORs; whereas depression disclosed lower ORs, 2.5 (1.5–3.9) for all headaches; 3.4 (2.0–5.7) migraine; 3.8 (1.8–8.3) chronic migraine, and 1.1 (0.4–3.7) for tension-type headache. These numbers supported the ideia that anxiety plays a more important role than depression in migraine risk. Moreover, the concept of a subthreshold diagnosis, (where patients fit all but one item for the full diagnostic criteria), being more linked to migraine than GAD diagnosis and depression, are reinforced by the findings in this study [23].

When we compare severe symptoms (e.g. when the aspect or symptom is felt every day), much higher odds are observed, particularly in anxiety, ranging from 24.4 to 49.2. “Not being able to stop or control worrying” (OR 49.2), “trouble relaxing” (25.9), “Feeling nervous, anxious or on edge” (25.4), “worrying too much about different things” (24.4) (Table 2). This “dose-response” observed by us is unique and displays that some subsets of patients with anxiety and depression display a much higher risk than what has been reported by other populational studies [24, 25]. It is noteworthy that not only excessive worry or tension, but the inability to control them was found to be more important. Sheftell and Atlas [26] mentioned losing the locus of control was a tipping point in migraine chronification.

The implication of not controling anxiety on a daily basis, as well as not easily relaxing, feeling anxious and experience daily excess in worrying should be better evaluated in migraineurs in both population and clinic settings. Not only in patients with a subthreshold, subclinical, partial, or subsyndromal psychiatric diagnosis, terms used synonymously to refer a clinical syndrome that does not fully meet the DSM or ICD diagnosis; but also in patients where those aspects can be found isolated, and should be taken in consideration in migraine management. The higher patients scored in the GAD-7 scale, the higher the severity of anxiety, and patients scoring higher than 7 (0–21) have shown a high sensitivity and specificity [27] for the GAD diagnosis. The item “Not being able to stop or control worrying” might be a good screening question to assess anxiety implication in migraine.

Detecting anxiety symptoms and implementing pharmacological and non-pharmacological treatments targeting those patterns could improve headache control and patients’ quality of life. Psychoterapies with cognitive-behavioural approach as well as physical and mental relaxation techniques may be usefull add on therapeutic strategies for migraine prevention. Although this has to be specifically tested in clinical trials, improvement in anxiety control is possibly the mechanism why behavioural treatments are efficacious in migraine treatment. The mechanisms behind the effect of preventive medications, antidepressants and/or neuromodulators, could be by improving anxiety-related abnormal physiological responses.

It is important to further understand the mechanisms behind the lack of control in anxiety, excess worry, and fear. Anxiety and fear-avoidance mechanisms have been linked to several pain disorders including primary headaches [22]. One may hypothesize a probable role of genetics, life events, psychological trauma, sleep, and cultural aspects.

Excess worry, fear, and other anxiety symptoms can be part of the migraine clinical spectrum. Irritability has been recognized as part of the prodrome [28], muscle tension a common finding in migraine and other headaches [29]. On the other hand, head pain may also be part of an anxiety clinical spectrum. A relevant discussion is whethere anxiety symptoms are part of the migraine spectrum, or vice-versa, and if their comorbidity is uni or bi-directional. As Merikangas first showed in 1990 and others confirmed [25], anxiety preceded migraine diagnosis, therefore early recognizing and treatment of anxiety symptoms in children and adolescentes may reduce the appearance of migraine in the future.

### Depression

The hallmark features of depression are emotional symptoms, hopelessness and sadness; nevertheless, the three highest scores found in our data were appetite, fatigue, and sleep disturbances, all physical symptoms.

Although not possible to define whether increase or decrease in appetite was occurring, we can suppose it was increase, based on epidemiological data in depressive disorders [30], and literature studying migraine and obesity [31]. Decrease in appetite may also be present, since nausea or vomiting are migraine symptoms. Our findings support a common biological mechanism between migraine and depression, through the hypothalamus, since it plays an important role in apetite, fatigue and sleep function. A hypothalamic dysfunction can be hypothesized as a link between both conditions.

The most related depression symptoms found: appetite, fatigue, and poor sleep may be overemphasized in migraine patients because they can be part of the migraine clinical picture [32, 33]. In addition, lack of concentration is commonly part of the migraine attack and is found interictally [34]. It is unlikely a migraine patient, at least near an attack, present without fatigue, apetite change, sleep problems, lack of concentration, or lack of pleasure. Feeling sad, although obviously expected during pain, was found to be less common, as low self-esteem and death thinking. One may speculate how valid and specific is depression diagnosis in migraine patients, simply applying DSM criteria and scales may not define the disorder, a reappraisal of both epidemiological and clinical data regarding depression and migraine comorbidity is necessary, previously published numbers could be affected by depression physical symptoms and migraine features, as well as case definitions, since studies varied accross DSM III, IV and V.

A diary study could answer how these overlapping symptoms are linked in headache disorders and psychiatric comorbidity, moreover, looking back into databases and subtracting anxiety and depression symptoms may help understanding the role of psychiatric symptoms in migraine.

Another key aspect is irritability, a symptom of the anxiety GAD-7 scale, but eventually more linked to depression than anxiety, particularly if we consider it as part of the bipolar spectrum. Becoming easily annoyed or irritable had an OR 3.8 (1.9–7.8) if experienced some days, 7.5 (2.7–20.7) more than half the days, and 22.0 (5.7–84.9) when experienced nearly every day. Irritability is also part of the Attention Deficit Hyperactivity Disorder clinical spectrum. ADHD overlap with anxiety clinical aspects, and can be a differential diagnosis. Four out of seven GAD items may be related to ADHD: trouble relaxing; feeling nervous, anxious or on edge; becoming easily annoyed or irritable; being so restless that is hard to sit still. ADHD is linked to the inability or lack of control [35].

Our paper supports the validity symptom-based concept, a relatively recent trend in mental health research and medicine [36]. By dissecting different elements in the two main disorder groups, anxiety and depression, and looking at their ranges, from mild to extreme manifestation, resulting in a more detailed, deeper understanding of psychiatric comorbidity with migraine.

Although inverting the traditional paradigm of starting with symptoms and moving towards a diagnosis, ask investigators to step back from pre-defined syndromes and focus on basic dimensions of functioning could be an important move in migraine comorbity research.

Strengths of our study are the robust sample size, comprehensive general population studied, not only a small set of young adults as seen in some seminal studies in the area (breslau and merikangas), migraine diagnosis were not self reported. Anxiety and depression were studied using detailed questionnaires, where sensitivity and specificity for a DSM diagnosis has been validated [27]. Limitations include its cross-sectional study design, where results are correlative and cannot address causality; absence of a full psychiatric diagnostic interview, where other mental health issues relevant to migraine comorbidity could be explore such as psychological trauma, ADHD, and the bipolar spectrum. The self-report nature of responses could be a limitation but most of the data on comorbidity was based on self-administered questionnaires.

Analyzing individual items from scales may be not considered as good practice because it lacks the reliability of the full scale, but this is the very concept we are challenging here. Anxiety and depression symptoms are overlapping and should be considered as a continuum spectrum of affective symptoms, especially in presence of pain disorders. Our objective was not to detect a psychiatric disease but understand within the aspects raised in each scale’s question, which are the most related to migraine, we think this is what our paper adds to the literature.

For further research, other psychiatric disorders, psychological and cultural aspects, beliefs, personality, and biological variables, from genetics to neuroimaging should be studied, as implications in public health and treatment outcomes. Studying other pain syndromes, other headache disorders, migraine subtypes, migraine chronicity, other chronic conditions, and their relation to anxiety and depression symptoms would improve our understanding in the field.

## Conclusion

Anxiety overall and its symptoms had a stronger association with migraine than depression. Hability to control worrying and relaxing are the most prominent issues in migraine psychiatric comorbidity. Physical symptoms in depression are more linked to migraine than emotional. A symptom-based approach helps clarifying migraine comorbidity and should be replicated in other studies.
